# Postpartum integration of infant vaccines and contraception through gender transformative programming: A cluster randomized trial in India

**DOI:** 10.1371/journal.pgph.0006403

**Published:** 2026-09-24

**Authors:** Sarah Averbach, Mohan Ghule, Gennifer Kully, Nicole E. Johns, Madhusudan Battala, Vedavati Patwardhan, Anita Raj, Shahina Begum

**Affiliations:** 1 Department of Obstetrics, Gynecology, and Reproductive Sciences, Division of Complex Family Planning, University of California, San Diego, La Jolla, California, United States of America; 2 Center on Gender Equity and Health, Department of Medicine, Division of Infectious Disease and Global Public Health, University of California, San Diego, La Jolla, California, United States of America; 3 Center on Gender Equity and Health, University of California, San Diego, India Office, Mumbai, Maharashtra, India; 4 Population Council, India, New Delhi, India; 5 Newcomb Institute, Tulane University, New Orleans, Louisiana, United States of America; 6 National Institute for Research in Reproductive and Child Health, Mumbai, Maharashtra, India; University of Washington, UNITED STATES OF AMERICA

## Abstract

Barriers to using contraception in India include norms that promote gender inequality by restricting mobility of women to reach health clinics. We evaluated whether PIVoT (Postpartum Integration of Vaccines and contraception through gender-Transformative programming) improves contraceptive use while enhancing autonomy in contraceptive decision-making. PIVoT delivers the full spectrum of contraceptive methods available along with gender-transformative counseling, linked with community-based infant vaccination. We conducted a cluster randomized trial; 20 *Anganwadi* Community centers were randomized to PIVoT or the standard-of-care. We included women 18 years and older, within 12 weeks postpartum, presenting for infant vaccination at community centers in rural Maharashtra, India. We measured contraceptive use (primary outcome) and reproductive autonomy after six months. From September 2023 to June 2024, we enrolled 293 women: 143 at intervention and 150 at control sites. Follow-up at six months was 95%. Any contraceptive use was more common for the control group at baseline (n = 57, 39.9% vs n = 93, 62.0%, p < 0.001) and for the intervention group at follow-up (n = 67, 48.9% vs n = 53, 37.3%, p = 0.051). There was a time-by-treatment effect for any contraceptive use (adjusted ratio of odds ratios 3.98, 95% CI 1.95-8.12). IUD use was slightly higher at six months in the intervention group (2.9% vs 0%, *p* = 0.04). Use of female-controlled methods (permanent contraception, pills, IUDs, injectables) was higher at six months among the intervention group (13.1% vs 6.3%, p = 0.04). Reproductive decision-making autonomy was higher among intervention participants at follow-up (2.02 vs 1.93, p = 0.02). The PIVoT intervention may increase contraceptive use postpartum, particularly female-controlled methods, while improving reproductive autonomy.

## Introduction

Using effective postpartum contraception allows women to prevent short inter-pregnancy intervals (fewer than 24 months between births), which are associated with an increased risk of maternal and infant morbidity and mortality [1–4]. Maternal and infant mortality in India are high, particularly in rural areas [5–7] and short inter-pregnancy intervals are associated with increased risk of maternal and infant morbidity [5,8–10]. However, less than half of women use contraception within the first year after delivery, and 50–75% of births do not follow the recommended interval [8,11–13]. Rural women are those with the greatest need; women living in rural India are less likely to initiate postpartum contraception compared to women living in urban areas [12].

Barriers to accessing and using contraception in rural India include gender norms that promote gender inequality by restricting the mobility of women to reach health clinics [14,15], limiting opportunities for male involvement in contraceptive decision-making [16], and facilitating male control over reproductive decision-making [17].

Under India’s public health system, a cadre of frontline health workers (FHWs), including nurses, provide community-based postpartum care for women and infants. India’s National Vaccination Program uses community-based delivery via FHW nurses for rural populations at monthly immunization camps incorporated into Village Health and Nutrition Days (VHNDs) [18]. In India, approximately 90% of infants receive the full vaccination series [19] and in rural areas 94% receive their vaccines from the public health system [20].

We hypothesized that linking community-based, gender-transformative family planning counseling (i.e., addressing gender norms that limit women’s autonomy in reproductive decision-making as part of family planning counseling) and contraceptive method provision with the existing infant vaccination services will reach more women than siloed unlinked services and will improve access to the full range of postpartum contraception and the ability to use family planning. Furthermore, gender-transformative programming has been shown to improve contraceptive use and control over family planning decision-making among women in rural India [21,22], but there is limited data among postpartum women specifically. Therefore, we developed the PIVoT intervention (Postpartum Integration of Vaccines and contraception through gender-Transformative programming) linking delivery of gender-transformative family planning programming and delivery of the full range of available reversible contraceptives with infant vaccination. The objective of this study was to pilot test PIVoT during infant vaccination visits in rural India and to assess the effect of the intervention on contraceptive method use and reproductive autonomy.

## Methods

### Ethics statement

Ethical approval for this study was obtained from the Institutional Review Boards of the India Council of Medical Research (#430/2020) and the University of California San Diego (#804819). Written informed consent was obtained from all participants prior to enrollment. This study is registered at clinicaltrials.gov (#NCT05732142).

### Study design

We conducted a two-arm cluster randomized controlled trial to evaluate the effect of the PIVoT intervention, compared to standard of care, on contraceptive use and women’s autonomy in contraceptive decision-making.

We randomized 20 geographic clusters within rural Maharashtra to receive the PIVoT intervention or the standard of care. Clusters were based on the subcenter, the catchment area of the *Anganwadi* Center (AWC), the most proximal level of community health care within India’s public health system, where monthly infant and child vaccination camps are held during Village Health and Nutrition Days (VHNDs).

### Participants

Between September 2023 and June 2024, women who had a vaginal or cesarean birth, presented with their infant for infant vaccinations, were 18 years and older, Marathi-speaking, and within 12 weeks postpartum were included. Women were excluded if they were using permanent female contraception or had an IUD in place, had a new pregnancy, or resided outside of the project area.

### Randomization and masking

Computer generated cluster randomization ensured that providers were consistent in offering either intervention or control services to participants, reducing risk for contamination. Auxiliary nurse midwives (ANMs) positions are specific to the AWC unless they are covering for a vacancy in a neighboring AWC. Neither participants nor research staff were masked to the treatment assignment, but control cluster participants and providers were not familiar with what the intervention entailed.

### Procedures

Baseline and Follow-up Surveys: Research staff obtained written informed consent and conducted baseline surveys in person in private settings; staff who were blinded to group assignment administered surveys and directly entered the responses into a secure electronic tablet. The surveys were conducted in the local language, Marathi, always prior to delivery of the intervention for those in the intervention group. Follow-up surveys were conducted six months after the baseline survey in person at the participant’s home or AWC (per their choice). Rarely, if a participant had migrated outside the study area, the survey was administered by phone (n = 4).

The PIVoT intervention: consists of an individual 30–40-minute counseling session with a participant and a trained ANM nurse immediately before or after an infant vaccination visit. Women could choose whether to include their husbands in the session. Following the session, participants could choose to initiate any available reversible contraceptive method that same day. The infant vaccine schedule provides multiple opportunities to offer family planning care to postpartum women and couples over the first four months after birth. Therefore, up to three additional counseling sessions were available on request with the participants, with or without their husbands, at subsequent infant vaccine visits. PIVoT was designed to provide person-centered contraceptive choice through an information exchange that allows for selection of a contraceptive method that meets the needs of the person or couple and includes the choice to use modern contraceptive methods, fertility awareness contraceptive methods, or no method. PIVoT materials include 1) gender-equity and family planning curriculum flip charts that cover basic family planning information and address gender norms and son preference (common in India); 2) contraceptive method index cards, which detail the characteristics, use, duration of action, effectiveness, side effects, and contraindications associated with each method available in the public health system; and 3) a provider training manual. Contraceptives were provided at no cost to the participant via the government supported family planning program. The option to initiate any methods including long-acting reversible contraceptives (LARC) methods in the community setting immediately after vaccination visits is not standard of care in this region; method initiation typically requires a visit to a primary health center.

The intervention’s gender-transformative counseling explores whether and how gender norms supporting male decision-making control over family planning and pressure from in-laws to have more children, particularly sons, may affect women’s reproductive autonomy. Additionally, the intervention highlights the value of male partner involvement in family planning. However, it also validates a woman’s choice to use her preferred contraceptive without disclosure to a partner if she chooses or has concerns about intimate partner violence or reproductive coercion (behaviors to promote unwanted pregnancy, such as interference with contraception, and/or behaviors to promote pregnancy when it isn’t wanted as “contraceptive sabotage”). The intervention further supports male engagement in family planning, inviting participants to return for optional follow-up PIVoT sessions at subsequent infant vaccination visits either alone or with their partner.

The counseling tools were developed for this study, initially adapted using Government of India resources and an adapted version of the Population Council’s Balanced Counseling Strategy [23]. We borrowed gender-transformative approaches from two evidence-based family planning counseling interventions which include a focus on both reproductive autonomy for women and inclusion of men [24,25] and tools to address reproductive coercion within family planning counseling. These interventions had not been applied to postpartum couples, prior. If a contraceptive method was desired, it was provided in a private space by the ANMs administering vaccinations.

Subsequent to designing the intervention, we completed a qualitative assessment among patients and clinicians. Both perceived the model to be feasible and acceptable for potential use in their communities [26]. We gathered input from local stakeholders on how to adapt and implement the intervention to ensure fit with local resources, priorities, and processes and adapted our intervention based on the input received.

Control: the control group were offered government family planning brochures, and condoms by the ANMs during infant vaccination visits and they were referred to the affiliated public health services for further family planning care according to the standard of care. Counseling and free contraceptive methods are offered at the health centers including all methods available to the intervention group.

### Outcomes

Primary outcome included any contraceptive use (modern contraceptive methods, lactational amenorrhea (LAM), abstinence, fertility awareness methods) and modern contraceptive use [27] (permanent (“sterilization”), intrauterine, injectable, or emergency contraception; implants, combined and progestin-only pills, and barrier methods) and type of contraceptive use; we validated the responses of women who reported LAM if 1) participants were exclusively breastfeeding, 2) remained amenorrheic since birth, and 3) were less than 6 months postpartum [28]. Secondary outcomes included reproductive autonomy, assessed via the validated 14-item Reproductive Autonomy Scale (RAS) [29]. The RAS captures constructs related to a woman’s ability to achieve her reproductive intentions across three subscales, namely freedom from coercion, communication, and decision-making. Each subscale is scored as an average of its included items (ranging from 1-4 for the freedom from coercion and communication subscales and 1–3 for the decision-making subscale), with higher score representing greater autonomy. We also created a single RAS score which as a sum of subscale scores, ranging from 1-11, with higher score again representing greater autonomy. We assessed future pregnancy intention using the validated 14-item Desire to Avoid Pregnancy (DAP) Scale that has been shown to be acceptable for use in India using psychometric testing [30]. The DAP is scored as a single average across all items. with scores ranging from 0 to 4 and higher scores reflecting greater desire to avoid pregnancy.

Covariates: We assessed age, age at marriage, education, religion, caste, parity, having a living son (because of son preference and it’s potential effect on contraceptive use in India [31], co-residence with mother-in-law, past-year employment, and below poverty line (BPL) card ownership [a proxy for low-income].

### Statistical analysis

We estimated that a total of 286 women would be needed to detect a difference of 15% or greater in modern contraceptive [27] use between the two groups at six months postpartum, with 80% power, assuming a two-sided alpha of 0.05, and a 14% baseline rate of modern contraception use other than permanent contraception [32], taking into account an estimated 10% loss to follow-up and assuming an intraclass correlation coefficient (ICC) of 0.01 to account for the cluster-randomized design. Assumptions for clustered effects for the study site were derived from our Counseling Husbands and wives to Achieve Reproductive Health and Marital Equity (CHARM2) study, which was conducted in a similar age population in the same region but not in any of the same subcenters [22].

First, we assessed descriptive statistics, including baseline demographics and outcomes at follow-up by treatment. Next, we assessed intervention effects on current use of any contraceptive, current modern contraceptive use, and reproductive autonomy outcomes using an intent-to-treat design and difference-in-differences logistic or linear regression approach depending on outcome type, including mixed-effects models with nested random effects to account for cluster randomization. We constructed minimally adjusted models, accounting only for time (baseline, 6-month follow-up), treatment status, and time-treatment interaction. We then constructed fully adjusted models, including baseline demographic characteristics as fixed effects determined *a priori* based on established associations with contraceptive use. Covariates were assessed for multicollinearity; none was observed and all were retained. All models, unadjusted and adjusted, included woman nested within subcenter as random intercepts to account for clustering.

We assessed pregnancy and pregnancy intention during follow-up. As pregnancy was uncommon (only one woman reported pregnancy) we did not analyze pregnancy further. We used mixed effects linear regression to assess potential intervention effects on pregnancy *intention* using the same model specifications noted above.

Finally, to assess feasibility, acceptability and appropriateness of the intervention in this context, we also conducted basic frequency responses to the validated acceptability (AIM), appropriateness (IAM), and feasibility (FIM) of intervention measure [33] among women in the intervention group.

Significance was set at p < 0.05 for all comparisons including odds ratios (ORs), ratio of odds ratios (ROR), adjusted odds ratios (aORs), and adjusted ratios of odds ratios (aRORs); 95% confidence intervals (CIs) are reported throughout. All analyses were conducted using STATA 18.0. The CONSORT extended guidelines were used for reporting [34].

## Results

**Fig 1:**
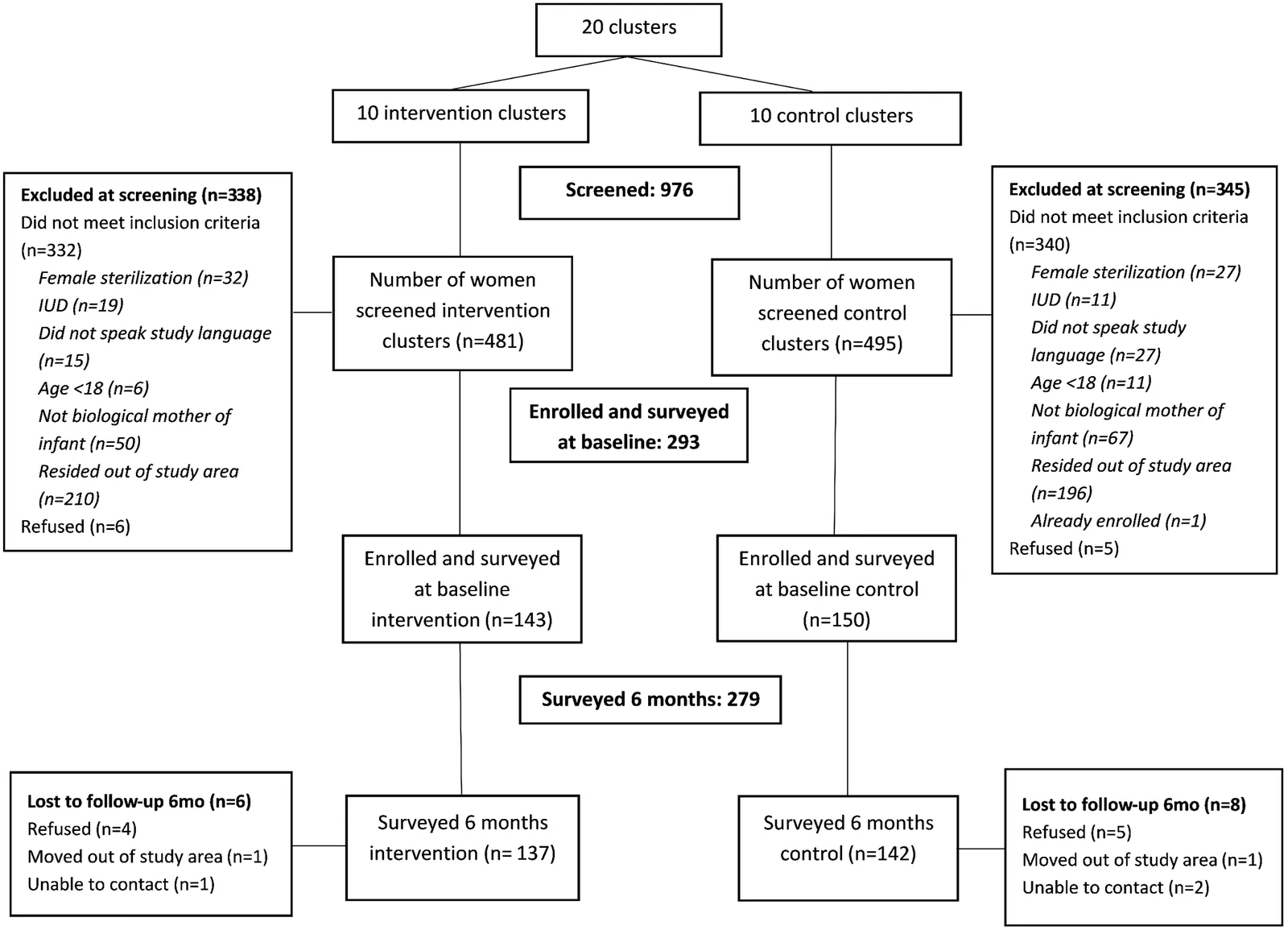
Consort Diagram the PIVot study.

From September 2023 to June 2024, we approached 976 women who presented with infants for vaccination visits. For 117, the infant’s grandmother, not mother brought the infant; of the 859 mothers, 555 did not meet inclusion criteria (due to residing out of the study area (n = 406), having undergone permanent female contraception (n = 59), not speaking the study language (n = 42), having an IUD (n = 30), being less than 18 years old (n = 17), and having already enrolled (n = 1)) and 11 declined to participate. The remaining 293 were enrolled (Fig 1): 143 (48.8%) were at cluster sites randomized to the intervention and 150 (51.2%) to clusters in the control group. At six month follow up 14 (4.8%) were lost to follow-up; this did not differ by treatment status (4.2% (n = 6) intervention vs 5.3% (n = 8) control, p = 0.65). Participant characteristics were similar between the groups (Table 1).

**Table 1:** Baseline characteristics of participants in the PIVoT study (n = 293).

	Overall (n = 293)	Control (n = 150)	Treatment (n = 143)
**Age (years)**	25.13 (4.62)	24.73 (4.61)	25.54 (4.60)
**Age at first marriage (years)**	20.73 (3.38)	20.63 (3.55)	20.84 (3.19)
**Ever attended school**			
No	6 (2.0)	4 (2.8)	2 (1.3)
Yes	287 (98.0)	148 (98.7)	139 (97.2)
**Religion**			
Hindu	272 (92.8)	143 (95.3)	129 (90.2)
Muslim or Buddhist	21 (7.2)	7 (4.7)	14 (9.8)
**Caste**			
General caste	181 (61.8)	88 (58.7)	93 (65.0)
Scheduled caste, scheduled tribe, other backwards class	112 (38.2)	62 (41.3)	50 (35.0)
**Parity**			
1	158 (53.9)	85 (56.7)	73 (51.0)
2	108 (36.9)	54 (36.0)	54 (37.8)
3+	27 (9.2)	11 (7.3)	16 (11.2)
**Has living son**			
No	113 (38.9)	53 (35.3)	61 (42.7)
Yes	179 (61.1)	97 (64.7)	82 (57.3)
**Lives with mother-in-law**			
No	63 (21.5)	43 (28.7)	20 (14.0)
Yes	230 (78.5)	107 (71.3)	123 (86.0)
**Worked in last 12 months**			
No	266 (90.8)	142 (94.7)	124 (86.7)
Yes	27 (9.2)	8 (5.3)	19 (13.3)
**Family has BPL card**			
No	219 (75.0)	114 (76.0)	105 (73.9)
Yes	73 (25.0)	36 (24.0)	37 (26.1)

### Any contraceptive use

Any contraceptive use decreased over the study period for the total study population, from 51.2% (n = 150) at baseline, to 43.0% (n = 120) at six months. Any contraceptive use was more common for the control group at baseline (n = 57, 39.9% intervention vs n = 93, 62.0% control, p < 0.001) and for the intervention group at follow-up (n = 67, 48.9% intervention vs n = 53, 37.3% control, p = 0.051) (Table 2). The decrease in overall contraceptive use, the difference in any contraceptive use between groups at baseline, and the larger drop over time observed for the control group were all driven by the use of LAM at baseline which, by definition, could no longer be in use at follow-up due to the time-constrained nature of the method (LAM is considered a contraceptive method only up to 6 months postpartum). At baseline, 108 (36.9%) women were exclusively using LAM, 41 (28.7%) intervention and 67 (44.7%) control participants (p = 0.01); 72% of all contraceptive use at baseline was exclusive LAM use (equivalent across groups, 72% intervention and 72% control, p = 0.99). In the unadjusted difference-in-difference model, there was a large and statistically significant time-by-treatment effect for any contraceptive use at six month follow-up (ROR 4.24, 95% CI 2.09-8.60); this effect was similar in the adjusted model (aROR 3.98, 95% CI 1.95-8.12).

**Table 2:** PIVoT study outcomes by treatment condition at baseline and 6-month follow-up (n = 293).

		Baseline		6-month follow-up		
	Control	Treatment	p-value	Control	Treatment	p-value
Observations	(n = 150)	(n = 143)		(n = 142)	(n = 137)	
**Any modern contraceptive method use,** N (%)	14 (9.3%)	14 (9.8%)	0.89	46 (32.4%)	55 (40.1%)	0.18
Any contraceptive method use, N (%)	93 (62.0%)	57 (39.9%)	<0.001	53 (37.3%)	67 (48.9%)	0.051
Any contraceptive method use, other than LAM, N(%)	26 (17.3%)	16 (11.2%)	0.13	53 (37.3%)	67 (48.9%)	0.051
**Contraceptive use, by type**^**1**^, N(%)						
Condom	11 (7.3%)	14 (9.8%)	0.45	38 (26.8%)	37 (27.0%)	0.96
Combined Pill	3 (2.0%)	0 (0%)	0.09	1 (0.7%)	0 (0%)	0.33
Progestin-only Pill	0 (0%)	0 (0%)	--	1 (0.7%)	0 (0%)	0.33
IUD^2^	0 (0%)	0 (0%)	--	0 (0%)	4 (2.9%)	0.04
Injectable	0 (0%)	0 (0%)	--	2 (1.4%)	7 (5.1%)	0.08
Rhythm	1 (0.7%)	1 (0.7%)	0.97	2 (1.4%)	2 (1.5%)	0.97
Withdrawal	16 (10.7%)	6 (4.2%)	0.04	6 (4.2%)	13 (9.5%)	0.08
LAM	75 (50.0%)	47 (32.9%)	0.003	--	--	--
Permanent Female Contraception	--	--	--	5 (3.5%)	7 (5.1%)	0.51
**Reproductive Autonomy Scale**, Mean (SD)	8.53 (0.97)	8.60 (1.03)	0.57	8.23 (0.91)	8.42 (0.99)	0.10
Decision-making Subscale, Mean (SD)	2.00 (0.27)	2.00 (0.30)	0.84	1.93 (0.30)	2.02 (0.33)	0.02
Coercion Subscale, Mean (SD)	3.26 (0.44)	3.30 (0.46)	0.49	3.10 (0.37)	3.17 (0.39)	0.11
Communication Subscale, Mean (SD)	3.28 (0.47)	3.30 (0.48)	0.67	3.18 (0.48)	3.22 (0.48)	0.41
**Desire to avoid pregnancy (DAP) score**, Mean (SD)	2.89 (0.33)	2.77 (0.51)	0.02	2.69 (0.81)	2.88 (0.66)	0.03

^1^Women could indicate use of more than one method (N = 20 women at baseline and N = 5 women at 6-month follow-up did so).

^2^Contraceptive use was assessed as ‘use since birth’ at baseline and ‘current use’ at follow-up. Two women who reported they had received IUDs immediately postpartum but who had expelled it by the time of baseline survey were considered non-users of IUD at baseline in analyses.

As a post-hoc sensitivity test, we examined any contraceptive use excluding LAM use; this did not differ between groups at baseline (n = 16, 11.2% intervention vs n = 26, 17.3% control, p = 0.13). In unadjusted and adjusted difference-in-difference models excluding LAM, we observed a significant time by treatment effect on use (unadjusted ROR 3.16, 95% CI 1.27-7.84; aROR 1.95, 95% CI 1.19-7.33).

### Current modern contraceptive use

Current modern contraceptive use increased over the study time period for the total study population, from 9.6% (n = 28) at baseline, to 36.2% (n = 101) at six months. Modern contraceptive use was equivalent at baseline (n = 14 in both groups, 9.8% intervention vs 9.3% control, *p* = 0.89), and slightly higher (though this did not reach statistical significance) among intervention participants at six month follow-up (n = 55, 40.1% intervention vs n = 46, 32.4% control, *p* = 0.18). In unadjusted difference-in-difference models, no significant time by treatment effects were present at six-month follow-up (ROR 1.50, 95% CI 0.51-4.36) (Table 3). The magnitude and significance of findings were similar in adjusted models (AROR 1.41, 95% CI -0.48-4.15).

**Table 3:** Difference-in-differences logistic and linear regression time-treatment interaction effects, assessing PIVoT impact on modern contraceptive use, any contraceptive use (n = 293).

Outcome	Interaction	Unadjusted^1^			Adjusted^2^		
***Logistic regression models***		**ROR**	**95% CI**	**p-value**	**AROR**	**95% CI**	**p-value**
Current modern contraceptive use	Intervention # 6mo follow-up	1.50	[0.51-4.36]	0.46	1.41	[0.48-4.15]	0.53
Current use of any contraceptive method	Intervention # 6mo follow-up	4.24	[2.09-8.60]	<0.001	3.98	[1.95-8.12]	<0.001
Current use of any contraceptive method except LAM	Intervention # 6mo follow-up	3.16	[1.27-7.84]	0.01	2.95	[1.19-7.33]	0.02
***Linear regression models***		**β**	**95% CI**	**p-value**	**β**	**95% CI**	**p-value**
Reproductive autonomy (full scale)	Intervention # 6mo follow-up	0.124	[-0.160-0.407]	0.39	0.125	[-0.160-0.410]	0.39
Reproductive decision-making (subscale)	Intervention # 6mo follow-up	0.085	[-0.001-0.170]	0.05	0.082	[-0.003-0.167]	0.06
Reproductive coercion (subscale)	Intervention # 6mo follow-up	0.036	[-0.094-0.167]	0.58	0.035	[-0.096-0.166]	0.60
Reproductive communication (subscale)	Intervention # 6mo follow-up	0.025	[-0.119-0.170]	0.73	0.029	[-0.117-0.175]	0.70
Desire to avoid pregnancy	Intervention # 6mo follow-up	0.311	[0.126-0.497]	0.001	0.307	[0.120-0.495]	0.001

^1^Unadjusted models include time (baseline, 6-month), treatment, and time-treatment interaction, with individual nested within subcenter random intercepts to account for repeated measures over time and subcenter clustering.

^2^Adjusted models include same specifications as simple models, plus age, age at marriage, school attendance, religion, caste, parity, has living son, lives with mother-in-law, worked in past year, and BPL card ownership.

IUD use was slightly higher at six months among women in the intervention group (n = 4, 2.9% vs n = 0, 0%, *p* = 0.04). Use of highly effective methods (injectable contraception, IUDs, and permanent female contraception) at six months was higher among the intervention vs. control group (n = 18, 13.1% vs n = 7, 4.9%, p = 0.02). Female controlled methods of contraception (permanent female contraception, birth control pills, IUDs, injectable contraception) was higher at six months among the intervention group (n = 18,13.1% vs n = 9, 6.3%, p = 0.04).

### Reproductive autonomy

We assessed the effect of PIVoT on reproductive autonomy using the Reproductive Autonomy Scale (RAS). Reproductive decision-making (range 1–3, higher score representing greater decision-making) was equivalent at baseline between groups (2.00 in intervention and control, p = 0.84) and was higher among intervention participants at six months (2.02 intervention vs 1.93 control, p = 0.02).

### Desire to avoid pregnancy (DAP)

Average DAP score (range 0–4, higher score representing greater desire to avoid pregnancy) was lower among treatment than control participants at baseline (2.77 vs 2.89, p = 0.02) and higher among treatment than control participants at follow-up (2.88 vs 2.69, p = 0.03). There was a significant time by treatment effect on DAP in both unadjusted and adjusted models, suggesting greater increases in desire to avoid a pregnancy over time for the PIVoT group (coefficient 0.12, 95% CI 0.13-0.50; adjusted coefficient 0.31, 95% CI 0.12-0.50).

### Acceptability, appropriateness, and feasibility

Acceptability, appropriateness, and feasibility of the intervention were all high; among intervention participants at follow-up, all (100%) selected ‘agree’ or ‘strongly agree’ to all acceptability (AIM), 98.5% to the appropriateness (IAM), and 96.4% to feasibility items (FIM).

## Discussion

### Main findings

We found that women in the PIVoT intervention group were 12% more likely to use a modern contraception method than in the control group at six months postpartum. As the study was only powered to show a 15% difference, this effect was not statistically significant (p = 0.051) likely due in part to small sample size and lower than expected baseline contraceptive use. We observed an increased rate of using any contraception among intervention participants at follow-up when accounting for baseline differences in use, particularly LAM, supporting the potential effectiveness of the intervention. Additionally, we found high willingness to participate in the intervention among those recruited and eligible, and PIVoT participants reported positive experiences with the program and found it feasible and acceptable.

### Strengths and limitations

Strengths include the randomized design, low loss-to-follow-up, inclusion of all available contraceptive methods in the intervention delivery, and that the intervention was embedded in the local health system—optimizing potential for scale-up.

Limitations include the higher-than-expected rate of contraceptive use in the control group, driven by LAM. Additionally, selection bias is possible since participants willing to participate in trials may be more motivated to use contraception than those who decline. There is also the possibility of contamination from intervention to control. There were rare vacancies in control sites which were covered by ANMs working at intervention sites. They were asked not to deliver the intervention at the control sites. However, they may have inadvertently utilized their counselling training across sites. Additionally, misconceptions about the safety of initiating contraception prior to having a menses postpartum, may have limited fidelity to the study protocol and decreased initiation of effective methods of contraception when desired. Finally, women in rural India commonly receive postpartum home visits from FHWs, typically community health workers called ASHAs (Accredited Social Health Activist) but sometimes ANMs. At these home visits, the FHWs provide basic information about family planning and can distribute condoms. They are not trained to provide gender-transformative care, and they are not providing reversible contraceptive methods, or services linked to infant vaccination. Therefore, there is no reason to believe that home visits confounded the intervention effect. Among women who reported using a method at baseline, 76% reported they got information about that method from an ASHA at their home and 25% from an ANM at their home (could select multiple options). Rates for reporting each were equivalent between groups.

### Interpretation

These findings expand prior research indicating receptivity of postpartum women to family planning counseling linked with infant care, documenting that the approach can promote postpartum contraceptive use. Several studies demonstrated that contraceptive uptake increases [35–37], or short inter-pregnancy intervals decrease [38], after integrating family planning and vaccination services. However, linked family planning and infant vaccination interventions have not previously been designed to address gender norms and male engagement as barriers to contraceptive uptake (i.e., used a gender-transformative approach) which is what makes PIVoT novel.

We not only found a trend towards using contraception at a higher rate in the PIVoT group but they reported greater autonomy in contraceptive decision-making, as measured by the decision-making subscale of the validated RAS measure. Thus, PIVoT participants were more certain in their ability to use contraception, if they desired. These findings correspond with prior research documenting that gender-transformative family planning interventions not only increase contraceptive use, they increase women’s autonomy in reproductive decision-making [39]. We also observed an increase in the use of female-controlled contraceptive methods, suggesting increased empowerment in contraceptive decision making [39].

PIVoT is also unique among interventions integrating family planning and infant vaccination in its focus on including the full spectrum of reversible contraceptive methods available, even IUDs, despite the implementation challenges associated with delivery of IUDs in community settings. We found that use of highly effective methods (injectable contraception, IUDs, and permanent female contraception), as well as IUDs alone, were both slightly higher. In addition, qualitative interviews with nurses in the region revealed a greater level of interest in more effective methods than this data indicates but that participants were sometimes discouraged from starting effective contraceptive methods at vaccination visits due to misconceptions about postpartum contraception [17]. To address this, and to further increase likelihood of contraceptive uptake if desired, we propose to adapt the training curriculum in the future to supports the safe use of effective contraception methods by postpartum people as soon as they desire to use them. In addition, contraception implants have since become available in the area and will now add another method to the method mix. Therefore, future adaptations of PIVoT may have greater effectiveness due to including a broader method mix.

## Conclusion

We found that the PIVoT intervention may increase contraceptive use postpartum, particularly female-controlled methods, while improving reproductive autonomy. Therefore, an adapted PIVoT intervention has the potential to decrease unintended short interval pregnancies while supporting person-centered contraceptive choice. Larger studies with longer follow-up are needed to understand feasibility at scale, and effectiveness on pregnancy outcomes.
