# Supplementary material for: Postpartum integration of infant vaccines and contraception through gender transformative programming: A cluster randomized trial in India
Source: PLOS Glob Public Health. 2026 Sep 24;6(9):e0006403. doi: 10.1371/journal.pgph.0006403 (PMC13606928; doi:10.1371/journal.pgph.0006403)
Supplement: Supplementary file 1 [file pgph.0006403.s002.docx]

PIVOT response to reviewers 06_04_26

**Journal Requirements:**

1. Please amend your detailed Financial Disclosure statement. This is published with the article. It must therefore be completed in full sentences and contain the exact wording you wish to be published.

i. Please clarify all sources of financial support for your study. List the grants, grant numbers, and organizations that funded your study, including funding received from your institution. Please note that suppliers of material support, including research materials, should be recognized in the Acknowledgements section rather than in the Financial Disclosure. 
ii. State the initials, alongside each funding source, of each author to receive each grant. For example: "This work was supported by the National Institutes of Health (####### to AM; ###### to CJ) and the National Science Foundation (###### to AM)."
iii. State what role the funders took in the study. If the funders had no role in your study, please state: “The funders had no role in study design, data collection and analysis, decision to publish, or preparation of the manuscript.”
iv. If any authors received a salary from any of your funders, please state which authors and which funders.

***The ‘Funding’ statement within the manuscript has been updated accordingly (page 17).***

2. In the online submission form, you indicated that your data will be submitted to a repository upon acceptance. We strongly recommend all authors deposit their data before acceptance, as the process can be lengthy and hold up publication timelines. Please note that, though access restrictions are acceptable now, your entire minimal dataset will need to be made freely accessible if your manuscript is accepted for publication. This policy applies to all data except where public deposition would breach compliance with the protocol approved by your research ethics board. If you are unable to adhere to our open data policy, please kindly revise your statement to explain your reasoning and we will seek the editor's input on an exemption.

***We are in the process of ensuring that all codes are fully labeled, ensuring codebook is fully up to date, etc. The dataset will be upload to the DASH repository no later than 7/1/26.***

3. Please ensure that your Ethics Statement is available in its entirety at the beginning of your Methods section, under a subheading 'Ethics Statement'. It must include:

i) The name(s) of the Institutional Review Board(s) or Ethics Committee(s)

ii) The approval number(s), or a statement that approval was granted by the named board(s)

iii) (for human participants or donors) - A statement that formal consent was obtained (must state whether verbal/written) OR the reason consent was not obtained (e.g., anonymity).

vi) (for child participants) - Please update your Ethics Statement with confirmation that written informed consent was obtained from the parent/guardian of each participant under 18 years of age.

***We have updated the Ethics statement in the Methods section accordingly (page 17).***

4. Please provide separate figure files in .tif or .eps format.

***Figure 1 is now provided as a .tif file which has been confirmed to meet file requirements by the NAAS tool.***

5. We noticed that you used “data not shown” in the manuscript. We do not allow these references, as the PLOS data access policy requires that all data be either published with the manuscript or made available in a publicly accessible database. Please amend the supplementary material to include the referenced data or remove the references.

***We have edited the sentence to remove the reference to data not and added a citation accordingly (page 16).***

6. Tables should not be uploaded as individual files. Please remove these files and include the Tables in your manuscript file as editable, cell-based objects.

***Tables 1, 2, & 3 have been added to the manuscript as editable objects.***

**Reviewer #1**: This manuscript reports findings from a cluster-randomized controlled trial evaluating the impact of the PIVoT intervention on contraceptive use and women’s contraceptive agency. The statistical plan using regressions for obtaining effects is sound. Indeed, it is reassuring that the results remained consistent after adjustment for variables. Additionally, the figures and tables are clearly presented and well-prepared. However, the methodology section would benefit from additional detail on several key aspects as explained below, in particular about the sample size estimation.

The sample size estimation process should be described with greater clarity. For example, the manuscript should specify whether the estimation accounted for a design effect and for potential losses to follow-up. If these factors were not accounted for, as seems to be the case, the necessary sample size would likely exceed the figure of N=286 women. The manuscript already notes that the study was underpowered, with the sample size estimated based on an effect size of 15%. The manuscript must also acknowledge these other methodological limitations that contributed to the study's lack of statistical power, even though loss to follow-up was minimal.

***Thank you for highlighting the unclear wording initially used – we did indeed take into account the clustered study design via an assumed intraclass correlation coefficient (ICC) of 0.01; this was based on the observed ICC for modern contraceptive use in prior research in the study area using a similar geographic unit of clustering [subcenter]. We also accounted for loss to follow-up in our initial power calculation, assuming 10% loss to follow-up. The text regarding power has been clarified to read as follows (page 8-19):***

***“We estimated that a total of 286 women would be needed to detect a difference of 15% or greater in modern contraceptive use between the two groups at six months postpartum, with 80% power, assuming a two-sided alpha of 0.05, and a 14% baseline rate of modern contraception use other than permanent contraception, after taking into account an estimated 10% loss to follow-up and assuming an intraclass correlation coefficient (ICC) of 0.01 to account for the cluster-randomized design. Assumptions for clustered effects for the study site were derived from our Counseling Husbands and wives to Achieve Reproductive Health and Marital Equity (CHARM2) study, which was conducted in a similar age population in the same region but not in any of the same subcenters.”***

The methods section should provide a more detailed description of the instruments used for contraceptive agency and its evaluation.

***We have added additional detail regarding the measure used for the quantitative assessment of contraceptive agency, including number of items, subscales, and scoring. We have also modified wording in text to more accurately note that we have assessed reproductive autonomy specifically rather than using the term ‘contraceptive agency’.***

***“Secondary outcomes included reproductive autonomy, assessed via the validated 14-item Reproductive Autonomy Scale (RAS). The RAS captures constructs related to a woman’s ability to achieve her reproductive intentions across three subscales, namely freedom from coercion, communication, and decision-making. Each subscale is scored as an average of its included items (ranging from 1-4 for the freedom from coercion and communication subscales and 1-3 for the decision-making subscale), with higher score representing greater autonomy. We also created a single RAS score which as a sum of subscale scores, ranging from 1-11, with higher score again representing greater autonomy.***

The methods section should also provide a clear and comprehensive description of the reproductive autonomy scale and DAP scores, rather than deferring this information to the Results section. Including these details in the Methods will enhance clarity and help readers better understand the study's measurement tools.

***We have now added additional detail regarding the reproductive autonomy scale as outlined above and the DAP scale to the methods section as follows (page 8):***

***“We also assessed future pregnancy intention using the validated 14-item Desire to Avoid Pregnancy (DAP) Scale that has been shown to be acceptable for use in India using psychometric testing. The DAP is scored as a single average across all items. with scores ranging from 0 to 4 and higher scores reflecting greater desire to avoid pregnancy.”***

The statement in lines 275 to 278 should be revised: the statement claims a marginally significant effect, but the confidence intervals (CI 0.95–6.33; adjusted 95% CI 0.89–5.98) do not support this interpretation.

***We agree with the reviewer and have revised the statement to remove any suggestion of significance as follows: In unadjusted and adjusted difference-in-difference models excluding LAM, we did not observe a significant time by treatment effect on use (unadjusted ROR 2.46, 95% CI 0.95-6.33; aROR 2.31, 95% CI 0.89-5.98) (page 12).***
